# The SEO effect. Mapping the optimized landscape around controversial policy issues in Italy

**DOI:** 10.3389/fsoc.2023.1144669

**Published:** 2023-02-23

**Authors:** Laura Caroleo, Giulia Giorgi, Camilla De Amicis

**Affiliations:** ^1^Department of Law, Economics and Sociology, University Magna Graecia, Catanzaro, Italy; ^2^Department of Social and Political Science, University of Milan, Milan, Italy; ^3^Independent Researcher, Milan, Italy

**Keywords:** Google search, search engine optimization, digital methods, electoral campaign, policy issues

## Abstract

This research investigates the use of search engine optimization (SEO) by political and non-political actors to amplify the visibility of search engine results. While there has been much theoretical speculation around the role of SEO techniques in boosting the ranking appearance of a website, few empirical studies have been undertaken to understand the extent to which SEO techniques are used to promote visibility online. This study takes Italy as a case study to map the information landscape around nine highly controversial issues during the Italian electoral campaign of 2022. Using a combination of digital methods and a tool detecting optimization in websites, our article aims at examining which actors employ SEO techniques to foster the circulation of their ideas and agendas around hot topics. Our analysis reveals that information channels, institutions, and companies are predominant, while political actors remain in the background. Contextually, data indicate that SEO techniques are employed by several recurrent editorial groups, company owners, and institutions. Ultimately, we discuss the impact of SEO techniques on the circulation and visibility of information around relevant policy issues, contributing to shaping and influencing public debate and opinion.

## Introduction

This research investigates which actors have employed search engine optimization (SEO) to amplify their visibility on search engine results concerning relevant policy issues. Search engine critique has long revolved around privileging mechanisms and how to identify them. While there has been much theoretical debate around the role of SEO in boosting the ranking appearance of a website, few empirical studies have been undertaken to understand who uses SEO techniques to promote their websites (Cui and Hu, 2011; Zilincan, 2015). In this study, we look at nine policy issues that have played a central role in the context of the 2022 Italian elections: abortion, euthanasia, work, LGBTQIA+, migration, taxes, healthcare, innovation and digitization, and environment, with the intent to explore which actors have mostly employed SEO techniques to promote their visibility online and, contextually, to make their voices heard about hot topics. For each topic, we formulated a list of keywords, which were identified through a manual search on the electoral programs of the four main political forces: center-right coalition, left coalition or PD party, Five Star Movement, and third pole.

The keyword list was used as queries on software that simulates a search on Google, extracts the top 20 website links, and outputs a probability score for optimization. Specifically, the tool analyzes the website HTML searching for SEO indicators, classifying websites into four classes according to these factors: definitely optimized, probably optimized, probably not optimized, and definitely not optimized (Lewandowski et al., 2021; Lewandowski and Schultheiß, 2022). For each query entry, the tool extracted the first 20 results returned by Google.

The results of our analysis indicate that SEO techniques are mostly employed by news channels, which are the most frequently occurring actors across the different topics in our dataset. Within this category, we have identified several recurring editorial groups such as *Condé Nast, Wolters and Kluwer*, and *Gedi*. Their widespread presence demonstrates how, through the use of SEO tools, news outlets can rise to prominence and even monopolize the information environment with respect to certain topics. Besides information channels, the first positions of Google search result pages were occupied by institutions and companies—especially in the context of healthcare and environmental issues. In our understanding, these actors employ SEO for advertising purposes, exploiting both the visibility granted by these techniques and the timely popularity of topics such as euthanasia, abortion, and climate change. In this scenario, it appears surprising the scarcity of political actors. Despite dealing with politically relevant issues, which played a relevant role in the debate surrounding the 2022 Italian political elections, the presence of leaders' and parties' websites is reduced to a minimum, hinting at the possibility for these actors to rely on other channels and other recommendations systems (e.g., social media) to make their voices heard. In conclusion, our piece reflects on the consequences and risks of using SEO to spread information about policy issues.

## Theoretical framework

According to *The Stanford Encyclopedia of Philosophy*,^1^ the term democracy refers “to a method of collective decision making characterized by a kind of equality among the participants at an essential stage of the decision-making process”.

In the transition from the direct democracy of the Athenian agora to the modern democracy defined as the “democracy of power,” even if the public square is no longer there, the need for the visibility of power remains and is satisfied through publicity and through the formation of a public opinion that is created through freedom of the press and political leaders making statements through the mass media (Bobbio, 2014).

As stated by Kelsen (1945), the will of the community in a democracy is always created through a continuous discussion between the majority and minority across a free examination of arguments for and against a given regulation of a matter. The discussion takes place not only in parliament but also, primarily, in political meetings, newspapers, books, and other means of disseminating public opinion. Insofar a democracy without public opinion is a contradiction in terms.

Political communication, which has become essential in electoral campaigns and beyond, is understood as “the exchange and confrontation of contents of public-political interest produced by the political system, the media system and the citizen-voter” (Mazzoleni, 1998, p. 34), has reached its third phase, the post-modern one, characterized by the widespread use of new technologies and mobile devices and by a more thematic, personalized and interactive consumption with massive use of the Internet as an alternative to television (Blumler and Kavanagh, 1999; Norris, 2000).

A well-organized online political communication can lead to the participation of a large number of people, reproducing it on an already large scale and benefitting from the synergies generated by the visibility that moves from the web to traditional media (Chadwick, 2013). In Italy, the case of the Five Star Movement is emblematic: a party that, without the use of the Internet, would not have been born and that has managed to establish itself precisely due to the web (Biorcio, 2013).

One of the fundamental aspects of democracy is the degree of freedom with which citizens vote based on consistent preferences. The use of new technologies, access to limited independence of information or fictitious and manipulated information, or their excessive personalization can lead to an increase in ideological biases, which could undermine voters' free and informed choices (Achen and Bartels, 2016; Suzor, 2019).

Precisely because of the Internet's infrastructure, search engines, and social media platforms have huge power in organizing information for their users, and the consequence is that not all choices become equal. Even the findability and visibility of some sites on the Google search engine follow different rules: some are highly indexed and rise to the top of the search results, while others are never indexed. The same happens with the visibility of political content on the Internet, which has a strong impact not only on the voice of the politicians themselves but also on the voters who come into contact with a certain type of information that can have significant effects on society and individuals, such as undermining a country's electoral process (Hindman, 2008; Pariser, 2011; Vaidhyanathan, 2011; Bucher, 2018).

Following the political agenda and the agenda setting,^2^ this research aims to study the use of SEO during the national electoral campaign in 2022 in Italy because “we look at Google results and see society, instead of Google” (Rogers et al., 2009, p. 49).

The role of public policy issues and how these are treated and covered by the media is very important during the election campaign and becomes even more decisive to the extent that voters perceive differences between candidates on political issues and vote on this basis (Alverez, 1998; Merrill and Grofman, 1999; Neufville and Barton, 2004). We refer to public policy issues as arguments involving a conflict over what the government should or should not do in this Googlization era.

Thus, in the informational society, where information development and transmission are the basis of productivity and power and where the source lies in the technology that generates knowledge, information processing, and symbolic communication, technological development, and its utilization acquire greater significance (Castells, 2002). Furthermore, in an economy based on the Internet, education, information, science, and technology become critical sources of value creation (Castells, 2001).

Information retrieval deals with the structure, analysis, organization, storage, search, and retrieval of information (Salton, 1968). Although its first definition dates back to the last century, its true meaning was best understood when Google was founded in 1998 with the idea of organizing the world's information and making it universally accessible and useful (Jones, 2013).

During a campaign, ensuring that content has good visibility in searches is vital for political actors. Over the years, practices, tricks, and habits have spread to enable sites to climb the rankings of search engine results pages (SERPs) (Giansante, 2014).

Google's SERP, formed by a list of organic content and a list of sponsored content, competing with each other to attract the attention of search engine users (Xu et al., 2012), is continuously updated and sees changing search results rankings to generate relevant results based on users' clicks (Clemons and Madhani, 2010; Baye et al., 2016) and presenting relevant information to users based on the search engine algorithms (Klatt, 2013).

The process of improving a website's visibility in organic search results is called SEO search engine optimization. The website is created so that it ranks well for the chosen keywords in the organic search results of the major search engines, the volume and quality of traffic to a website from search engines are improved organically due to the selected keywords, and unlike advertising, the traffic is organic and free (Chen et al., 2011; Kritzinger and Weideman, 2013; Iskandar and Komara, 2018).

It has already been shown not only that the ranking permanence of sites using SEO is much longer but also that users often tend to click more on organic listings than on paid ones, so the results at the top of the search results are more likely to be visited and clicked on, also because engine users over the years have been browsing fewer and fewer result pages (Jansen and Spink, 2009; Klatt, 2013; Panda, 2013; Baye et al., 2016; Dan and Davison, 2016).

Search engine optimization techniques can be distinguished into white hat techniques, i.e., accepted by search engines, and black hat techniques, which cause search engines to remove sites from the results until they are adjusted. White hat is the most legitimate way of implementing SEO to achieve high rankings and is based on Google's guidelines, following its algorithm and avoiding bad practices. In this way, growth is steady, gradual, and lasting. On the other hand, the black hat is an illegitimate way of ranking in the SERPs, is contrary to the guidelines, and finds fertile ground in the shortcomings and gaps in Google's algorithm.

Therefore, also following reports in the Italian press on the concerns of the Copasir^3^ (Parliamentary Committee for the Security of the Republic) about the risk of the public debate being polluted and distorted:

*Communicative strategies articulated in disinformation (all those practices of voluntarily creating and disseminating untrue or misleading information to deceive the recipient of the message), misinformation (all those practices of disseminating untrue information without the knowledge that it is false and, therefore, in the absence of the voluntariness of deceiving the recipient of the message) and media manipulation are established and spread; in data-driven strategies that make use of the ‘targeting' systems of large social media companies; in the use of trolling (the act of creating and disseminating online messages and comments of a violent or defamatory nature, prompting the recipient to an emotional response) or harassment directed at users' digital profiles; in mass reporting of content and accounts, indirectly exploiting the filtering systems of platforms*.

As mentioned earlier, studying SEO techniques during the 2022 electoral campaign that led to the election of the first woman as head of government in Italy is vital to understand how information was disseminated on topics that were (and still are) pivotal to the Italian political debate. Our research revolves around two research questions and two sub-questions, as follows:

1. Which actors appear in the first positions when searching for key policy issues of the Italian political elections?
  a. Which of them uses SEO?
  b. How relevant are political actors in this context?

## Methodology

Following the digital methods (Rogers, 2013, 2018) our empirical investigation focuses on nine social and political issues: abortion, euthanasia, work, LGBTQIA+, migration, taxes, healthcare, innovation and digitization, and the environment. These topics were identified through an exploration of the electoral programs of the most relevant parties (i.e., having at least the 5%) and coalitions competing in the 2022 Italian election. These are the center-right wing coalition (*Fratelli d'Italia, Lega Salvini Premier* and *Forza Italia*); *Partito Democratico* (PD); Terzo Polo (*Azione, Italia Viva*); and *Movimento 5 Stelle*. For each topic, we formulated a list of relevant keywords, either one word or more than one (see Appendix 1), which were manually extracted from said programs.

The keywords were then used as queries on software developed by the Hamburg University of Applied Sciences (HAW Hamburg), which simulates a search on Google, extracts the top 20 website links, and outputs a probability score for optimization. Specifically, the tool analyzes the website HTML searching for 32 SEO indicators, classifying websites into four classes according to these factors: definitely optimized, probably optimized, probably not optimized, and definitely not optimized (Lewandowski et al., 2021; Lewandowski and Schultheiß, 2022). We performed the search straddling the election day: 23, 24, and 25 September 2022. The output is a file including the following data: the input query, the link of the website, the position of the website in the ranking (from 1 to 20), and the probability of optimization.

We decided to limit our analysis to the first five results for each query, deeming them to be the most visually prominent on the Google result page and, thus, most likely to be seen (and visited) by users. The final dataset counts 1.335 websites, divided as follows: 55 for abortion, 45 for euthanasia, 230 for work, 130 for LGBTQIA+, 170 for migration, 160 for taxes, 205 for healthcare, 165 for innovation and digitization, and 175 for the environment. Afterward, we categorized the players appearing for each result into eight categories, according to the typology of actors owning the websites. Both researchers engaged in the coding process, which involved iterative rounds of individual coding and collective discussion. The categories were not decided *a priori* but emerged deductively from the data, following the principles of ethnographic content analysis (Altheide, 1987; Caliandro and Gandini, 2016). They are information channels, cultural institutions, research institutes, companies, political actors (including both pages of parties, coalitions, or individual leaders), institutions, and NGOs. In Table 1, we detail each category, providing a brief description of the type of actor it identifies. This specification appears to be particularly relevant in order to disambiguate overlapping terms and “lost-in-translation” types of nuances. Further analysis was directed at identifying recurring editorial groups, companies, and institutions behind the most relevant categories (in terms of numbers), which was carried out in a collaborative way by the authors, manually inspecting the websites included in the “news” category.

**Table 1:** Overview of the categories of actors behind the websites.

**Category**	**Description**
Information channels	It groups together national (e.g., *Il Sole 24 Ore S.p.A*. or *RCS S.p.A*) and local news outlets (*Citynews S.p.A*. or independent local ones) and also include independent newspapers (e.g., *ILPOST* or *Open.Online*)
NGOs	Non-governmental organizations such as *Amnesty International, Save the Children*, and Italian ones like *Associazione Luca Coscion*
Political actors	Websites of political parties and individual leaders. We have also included in this category official social media pages
Institutions	Within the definition institution, we have included: institutional sites of the government, institutional sites of the various ministries, the chamber of deputies and the senate, institutional sites of regions and municipalities, para-state and governmental bodies, national agencies, public health companies and public hospitals, and international organizations
Cultural websites	Encyclopedias (*Wikipedia, Treccani*) and vocabularies
Companies	Business-oriented companies, such as *Cisco System, Nestlè*
Research institutes	Universities or other research institutes
Self-employed workers	People who use their own sites to implement their customer base or for their personal branding

### Findings

**Figure 1:**
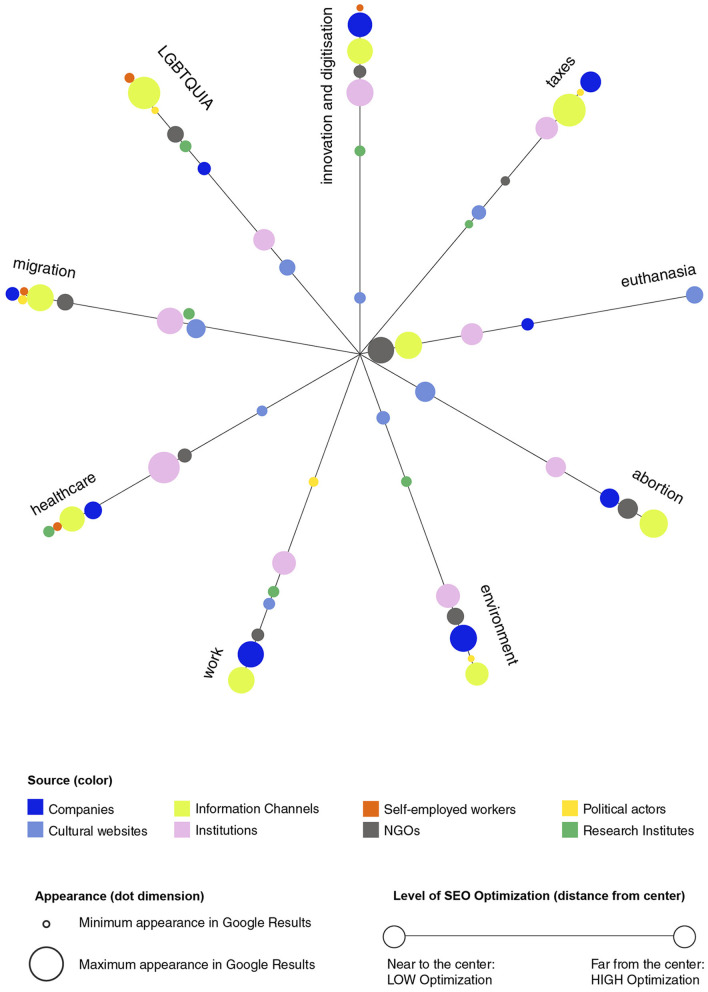
Optimization percentage per topic. Dot colors correspond to different actors (refer to legend), and their size corresponds to the frequency of occurrence in the dataset.

Looking at the typologies of actors present in the first five results, we notice that the majority of the websites are information channels, a broad category that comprises a variety of news and media outlets. Immediately following this category for frequency of occurrences in the dataset, companies and institutions are equally very present in our dataset (Figure 1). In the following sections, we look at the most prominent players across the issues analyzed, divided per topic, paying particular attention to recurrent actors, such as editorial groups (in the case of news outlets) or companies.

### Abortion

Website results connected to the topic of abortion present a majority of information sites, which amount to 36.36% of the actors. It is interesting to note that all these actors are optimized. A further look at this category, which constitutes the most prominent group in this topic (and many others, as we will see), reveals the presence of two editorial groups: *Condé Nast* and *Gedi*. The first is a U.S.-based global mass media company, whose media brands include Wired and Vanity Fair—both present in the dataset. The second is an Italian media conglomerate, owners of national newspapers such as *La Repubblica* and *La Stampa*. These are followed by NGOs and cultural websites (specifically *Wikipedia* and *Treccani* encyclopedia), totaling 16.36% of the websites. While their presence is the same, the optimization level for these two actors is the opposite: Most websites of NGOs are optimized, and *Wikipedia* results are classified as “most probably not optimized.” Then, we find companies and institutions, respectively 14.55 and 10.91%. Most of the websites included in these two categories result to be optimized for the most part.

### Euthanasia

Information websites constitute the predominant category (33.67%) of the first results outputted by the queries associated with the topic “euthanasia.” Furthermore, our analysis highlighted the presence of two editorial groups: *Mondadori* and *Wolters Kluwer*, both recurring four times (each) in the dataset related to euthanasia. *Mondadori*, the biggest Italian publishing company, appears because it is the owner of *My Personal Trainer*, a website spreading news and information around topics such as wellness, health, and nutrition. *Wolters Kluwer* is a Dutch information services company. It owns many of the information websites, and, as we will see, it is a recurrent player in other datasets as well. In this case, we find the site *Altalex*, the Italian newspaper of legal information.

Closely following the category “information channels,” we find sites of Non-governmental organizations (NGOs), counting 31.11%, such as *Fondazione Veronesi* and *Associazione Luca Coscioni*, an association advocating in favor of euthanasia. Other actors seem to have a minor impact (i.e., to be less frequently present) on the information landscape related to the topic: These include institutions, cultural websites, and companies. Looking at the overall optimization level of the players identified, we can observe that most of them have probably made use of SEO techniques to boost their online visibility. A notable exception is constituted by cultural websites, here represented by *Wikipedia*, which is always not optimized: This result is in line with the findings of other topics too.

### Work

As for work-related results, information channels (31.30%), sites, and companies (30.43%) represent—taken together—two-thirds of the actors in the dataset. Among the most influential editorial groups of the set, we find once again *Wolters Kluwer*, which recurs 11 times. In addition to Altalex, the other website present in the dataset belonging to this editor is Ipsoa, which—similarly to Altalex—describes itself as an online information service on taxation, financial statements, and accounting.

Another relevant group is constituted of institutions, which represent ~24% of the websites. Here, we find websites related to the Italian government, such as the page of the Work Department, the Ministero delle Imprese e del Made in Italy, INPS (the main entity of the Italian public retirement system), or unions' websites (e.g., CGIL). In addition to those, we find websites of local (i.e., regional) institutions, like ATS Sardegna, the web page of Sardinia region's healthcare system.

The remaining 10% of the results related to the study are fragmented into four categories: NGOs, cultural websites, research institutes, and political websites. Interestingly, political actors are the only category with a majority of non-optimized sites (60%), while for the other categories of actors, a significant proportion (if not the totality) of the results is classified as being optimized or most probably optimized.

### LGBTQIA+

The web results connected to LGBTQIA+ issues present little differences from the other topics presented so far, with respect to the categorization of actors. Once again, “information channels” appears to be the most frequently occurring type of actor present in the dataset, with almost half of the results being sorted into this group. When looking at the most recurrent editorial groups, we note the presence of already found ones—e.g., *Condé Nast, Gedi*, and *Wolters Kluwer*—along with new ones, such as *Gruppo Maggioli* and *Avvenire Editore*, which owns the Italian newspaper *Avvenire*, affiliated with the Catholic Church. Belonging to the first one is the website *diritto.it*, which is a resource for legal professionals, providing the latest information on the law.

Institutions and NGOs follow, counting 10 and 9.23%, respectively. Among the first ones, we found the official websites of the Italian Senate of the Republic and the Chamber of Deputies. Relevant NGOs identified in the dataset were *Amnesty* and *Pro Vita e Famiglia*, an association promoting and defending conservative views around (among others) sexual identities, marriage, and family. Cultural websites, companies, research institutions, self-employed workers, and political actors were identified as minor players: Individually taken, their presence does not reach 10%.

As for the optimization probability, all information websites have been classified as probably (or most probably) optimized. The same applies to political actors and self-employers. Other categories, such as companies, NGOs, and research institutions, show a majority of optimized results. On the other hand, institutions and cultural websites appear to be, for the most part, unlikely optimized.

### Migration

Consistently with the other topics, information (32.35%) is here too, the biggest category, immediately followed by institutions (31.18%). A closer inspection of the editorial groups reveals the already-seen *Gedi* group (here represented by the Italian newspaper *La Repubblica* and *Huffington Post*). In addition, we find the national public broadcasting company *RAI* (specifically Rai Scuola and Rai News) and the Italian national daily business newspaper *Il Sole 24 Ore*, which is owned by the Italian employers' federation *Confindustria*. According to the classification operated by the software we used, most news outlets have most likely employed SEO strategies to boost their visibility on the web.

After cultural institutions (14.12%) and NGOs (10%), the tail of the actors is constituted by companies, research institutes, political actors, and self-employed actors. It is interesting to observe that the categories of actors in this dataset are mostly optimized, with the sole exception of research institutes and cultural websites, which appear perfectly balanced. Some categories are instead entirely optimized (companies, political actors, and self-employed workers).

### Taxes

More than half (50.63%) of the websites connected to the topic “taxes” are information channels. Among them, we find several newspapers owned by *Network Digital 360*, a network of media outlets dedicated to the topics of digital transformation and entrepreneurial innovation. Examples of these outlets within this dataset include the website *Agenda Digitale, Corriere Comunicazioni*, and *Innovation Post*.

Institutions and companies come afterward, constituting, respectively, 21.25 and 17.50% of the total. Most websites of the category “institution” belong unsurprisingly to *Agenzia delle Entrate*, the Italian governmental agency that enforces the financial code of Italy. Among the most frequently occurring companies, we find portals of real estate advertisements for the sale and rental of apartments and houses, such as *Immobiliare, Idealista*, and *Immobili Ovunque*. Alongside those, there are websites for comparing rates for energy, online insurance, mortgages, and loans, telephony (e.g., *SuperMoney*), or job search portals (e.g., *Ti Consiglio Un Lavoro*).

Cultural institutions, NGOs, research institutes, and political actors represent the marginal players in the dataset: Aside from cultural institutions (6.88%), the other actors do not reach 2% of the total, individually. Taking optimization levels into consideration, we can see that the majority of all the categories appear to be optimized. Political actors are, once again, entirely optimized.

### Healthcare

The dataset of healthcare presents slightly different results when compared to the others since the ranking by frequency of institutions, and information is inverted. The institution is here in the lead, with 46.34% of the results. By inspecting the category, we found websites of many local sanitary systems (e.g., *ASL Roma, ASL AL*, and *Salute Lazio*), alongside national ones like, in particular, the *Istituto Superiore di Sanità* (ISS), the main center for research, control and technical-scientific advice on public health in Italy. Private institutions are also present: Among them, we mention private centers like *Futura IVF* and *IVI Italia*, both centers for medically assisted procreation, but also hospitals and RSA (e.g., Centro Residenziale Anziani “Umberto I”).

Conversely, information channels cover “only” 27.80% of the total. The biggest cluster of this group is composed of web pages from *Quotidiano Sanità* (QS), the online newspaper of health information, which is found 13 times in the corpus. Also quite frequently found is *Sanità 24*, the insert of the newspaper *Il Sole 24 Ore*, dedicated to health issues and the healthcare system.

The next most frequently found categories are companies (11.71%) and NGOs (6.34%). Within the latter, it seems relevant to note the presence of centers for research, like *The AIRC Foundation for Cancer Research* and the *Cystic Fibrosis Research Foundation (FFC)*. Cultural websites, research institutions, and political actors take, once again, the last positions—with <5% each. Apart from cultural institutions, which appear to be mostly unoptimized, the optimization level of the remaining categories of actors have definitely used SEO techniques to increase the visibility of their web pages: This appears evident in the case of information channels, and companies that are almost entirely classified as optimized.

### Innovation and digitization

The results related to the topic “innovation and digitization” present some novelty with respect to the ranking of the categories too: Here, information channels (25.45%) come third, after institutions (31.52%) and companies (30.91%). Looking closely at the institutions present in the dataset, it is worth mentioning that most sites belong to the Italian Data Protection Authority (*Garante per la protezione dei dati personali*), which is the supervisory authority responsible for monitoring the application of the General Data Protection Regulation (GDPR). Other institutional websites belong to different government departments, like the Department for Digital Transformation (*Dipartimento per la Trasformazione Digitale*), involved in defining strategies for modernization through digital technologies, or the Ministry of Enterprises and Made in Italy (*Ministero delle Imprese e del Made in Italy*).

Among the companies, the dataset features the U.S.-based multinational *Cisco*, the Italian telecommunications company *Tim* and Internet providers like *Ultranet*. The remaining categories (i.e., NGOs, cultural institutions, research institutes, and self-employers), taken individually, do not reach 5%. Political actors, like in many other cases, are absent.

Similarly to what we have observed for the “healthcare” dataset, all categories of actors appear to be optimized, with the sole exception of cultural institutions, which have a majority (~80%) of non-optimized websites.

### Environment

As for the results obtained from the search queries related to the environment, the first position is taken by companies (32.57%): Unsurprisingly, most of them are gas and electricity suppliers, such as *A2A, Enel, ENI*, and *VIVI Energia*. Other recurrent players are *Octovo*, a marketplace managing a network of energy installers, and *Selectra* and *Papernest*, web companies specializing in the comparison of electricity, gas, and Internet offers. In addition to those, we also found companies selling products such as *Colgate-Palmolive Company* and *San Pellegrino*.

Companies are followed by institutions (24.57%), among which it is possible to find both European (i.e., the site of the European Parliament) and national realities (like the already encountered *Agenzia delle Entrate*, the *Ministry of Sustainable Infrastructures and Mobility*, and the *Ministry of Health*). Very numerous are then the websites of local institutions: In the dataset, we identified sites of different municipalities and regions (Umbria, Veneto, Piemonte, Emilia-Romagna, the Metropolitan City of Milan, and Turin).

Similar to the topic innovation and digitization, the category information channel takes third place (22.86%) for frequency of occurrences. Here, no editorial group appears to predominate the others: Among the most frequently occurring ones, we find again *Wolters Kluwer* (with the websites Ipsoa and Altalex), *Network Digital 360* (Innovation Post), and *National Geographic*.

As seen for many other issues, NGOs, cultural institutions, research institutes, and political actors are found in the last positions of this ranking. Finally, the optimization level does not present innovation with respect to what has been observed so far, with a majority of optimized websites, especially in the category of “information channel,” where the totality of the pages has most likely employed SEO techniques.

### The role of politics

The almost total absence of political actors is perhaps the most relevant finding of our analysis. As a matter of fact, only five out of nine of the considered issues report the presence of political actors: work, migration, environment, tax, and LGBTQIA+. Moreover, the category “political actors” always takes the last position of the players, ranked for the frequency of occurrences: In fact, political actors appear three times in work-related results, three times in the migration dataset, and one time each for the remaining searches—environment, tax, and LGBTQIA+.

Nonetheless, we can observe that political actors have a prominent role with respect to their position in the page result ranking. In work, for instance, the first result output by the query *aiutiamo i lavoratori* (let us help the workers) is the official website of the Democratic Party *(Partito Democratico)*, while the website of Pietro Ichino, senator for the Democratic Party, appears as the first result for the query “lotta al precariato” and third when searching for “riduzione dell'orario di lavoro a parità di salario.”

In addition to websites, social media pages were found in the datasets: The official Facebook page of Nicola Zingaretti, former Democratic Party secretary, appears as the first result when querying for *Italia più umana e sicura*. The Twitter account of Matteo Salvini and the Twitter account of his party, Lega Salvini Premier, feature in the second and third position on the page result for *stop agli sbarchi*. The three remaining occurrences of political actors are as follows: The official Facebook page of current Prime Minister Giorgia Meloni, who appears as a third result for the query *no al matrimonio tra persone dello stesso sesso* (topic LGBTQIA+); the Five Star Movement official website, second result of the Google search *ridurre l'impatto del trasporto merci* (environment); and Massimo Ungaro's website (*Italia Viva* party), in third position for the query abbassare tasse sul lavoro (Taxes).

Looking at the optimization probability level, we can observe that—with the sole exception of the website of the Democratic Party—the rest of the political actors have been classified as being probably optimized. In particular, Matteo Salvini Twitter account, Lega Salvini Premier Twitter account, and Zingaretti's Facebook page have a high probability of having implemented SEO techniques (i.e., are classified as “most probably optimized”).

## Discussion

The article investigates the results outputted by Google search queries connected to nine key policy issues of the 2022 Italian electoral campaign, with the purpose of understanding which actors use SEO techniques to raise their visibility on the web. Our analysis revealed that information channels, institutions, and companies appeared most frequently in the first positions of the result page, whereas research institutes, cultural websites, and political actors were less significantly present. We also found that, with few exceptions (e.g., cultural websites), these categories of actors have most likely implemented search engine optimization strategies to climb to the top of the Google search result page.

From a global perspective, information channels are the predominant actors throughout the dataset. More specifically, we have identified the presence of large editorial groups, such as *Network Digital 360, Maggioli Group, Wolters Kluwer, Gedi Group, Condé Nast, Società Editoriale Fatto Quotidiano, Il Sole 24 Ore*, and *Mondadori Group*. It is worth noting that these actors usually traverse more than one topic: for instance, the *Wolters Kluwer* group appears in the results from queries connected to work, euthanasia, LGBTQIA+, and environmental issues. It is interesting to note that many of these websites are segment outlets, spreading information related to specific topics such as the legal one (e.g., *Altalex*), newspapers for accountants, medical doctors, and nurses (e.g., *Quotidiano Sanità*). Most importantly, the analysis of the optimization level has shown that large editorial groups are associated with a high probability level of SEO usage.

Another relevant finding regards the presence of a sheer variety of local media outlets alongside national newspapers. This is, too, partially connected to the presence of large editorial groups relying on SEO techniques to boost the visibility of their media products. For example, *CitynewsSPA* is one the largest companies, which owns many local newspapers contained in our datasets, such as *Milano Today, Roma Today*, and others. These findings appear to be consistent with recent trends, showing an uptick in the sales of local newspapers.^4^ On this note, research on digital journalism explored the different strategies of value creation implemented by local newspapers in the transition from print to digital environments. Drawing from our results, we might argue that local newspapers, especially those owned by large editorial groups, implement SEO techniques to carve out a relevant role in the digital informational space (Ragnhild, 2021).

While informational channels are predominant in most topics, healthcare and the environment appear dominated by institutions and companies, respectively. As for the topic of environment, it is interesting to observe that many companies selling energy and gas (e.g., *Enel, A2A*, and *Eni*), or that provide renewable energy services or connectivity, use SEO techniques not only for their own institutional sites but also for their sites that pretend to be publications in support of climate change: Some examples include news about the circular economy, clean, or renewable energy, the ecological transition, energy efficiency, sustainable mobility, and green tech. In reality, these appear to be cases of house organs to actualize “greenwashing” strategies. It is not uncommon for companies to “invest in green marketing communications, to be perceived as eco-friendly and socially engaged” more than they actually are (de Freitas Netto et al., 2020). Digital communication and online marketing have contributed to the rise of the phenomenon of greenwashing (Gräuler and Teuteberg, 2014; Adi, 2018). Our analysis demonstrates that different companies have relied on SEO techniques as a communication strategy in order to build and promote their public image of eco-sustainable, further spreading the circulation of the phenomenon of greenwashing.

Similarly, the massive use of SEO by clinics and private practices could be seen as a marketing strategy put in place to increase the visibility of these facilities. On a broader level, this tendency may be connected to the rising popularity of Italian private clinics due to the issues connected to the public health system (e.g., prolonged waiting periods, inferior quality of the service provided, and geographical inequalities) (Cioffi, 2021). In this sense, private clinics and nursing homes, among others, may invest in the use of SEO to boost their searchability by potential patients. In doing so, these facilities would benefit from the exposure granted on the one hand by SEO techniques and by the popularity of these topics for advertising purposes.

More observations can be made with respect to the marginal role played by websites and social media pages of political actors in the context of this work. As our study points out, politics is almost absent from the collected dataset. This finding is quite surprising if we consider that all nine topics are politically relevant and that we extracted the keywords employed for the query from political programs. Adding to the fact that the data collection was performed in the days leading to the elections, we expected political actors to be significantly more present in our datasets in terms of number and variety. While finding a cause for this evident absence falls beyond the scope of the present research, we may hypothesize that political actors do not use SEO on social media pages, as they believe that users will find their account through the platform and not search engine results.

In conclusion, our study provides the opportunity to reflect on the impact of SEO techniques in shaping the informational landscape regarding controversial policy issues, like the ones considered here. This may be relevant to analyze how SEO determines the consumption pattern of the digital population and information has become capital. As Castells (2007) argued, “media have become the social space where power is decided.” The emergence of a networked digital space where information flows has significantly changed power relationships in the distribution of news. According to the scholar, central hub(s), whose ultimate purposes can be undisclosed to users, provide and control the flow of information (Castells, 2001). Similarly, in our dataset, such a role is played by large companies and editorial groups: The extensive use of optimization enacted by these players can significantly undermine the variety of the information ecosystem found online, boosting the visibility of a small percentage of actors, to the detriment of variety of opinions and sources.

The present research comes with several shortcomings, such as the limited time and geographical reach (limited to just the Italian case), which may have undermined the generalizability of the results obtained. Future research may expand the scope of the research, scaling the analysis to other geographical areas or producing a longitudinal study. Despite its limitations, however, we argue that the present study contributes to the ongoing debate on SEO and its consequences with a previously overshadowed empirical perspective. More specifically, our findings do not only shed light on the type of actors that may be most interested in employing SEO but also on the underlying interests that push these actors to implement these techniques. Ultimately, it provides initial reflections of the extensive and unregulated use of SEO on the consequences on users' informational diet, which, unbeknownst to the web population, may be less varied and polyvocal than expected.

## Data availability statement

The raw data supporting the conclusions of this article will be made available by the authors, without undue reservation.

## Author contributions

The whole research is a result of intense collaboration among the authors LC and GG. CD contributed to the data analysis and data visualization. All authors contributed to the article and approved the submitted version.

## Footnotes

^1^ https://plato.stanford.edu/entries/democracy/

^2^We consider Political Agenda as the list of topics or problems that government officials, and people outside government closely associated with these officials, pay attention to at a given time; Agenda Setting is the process of defining these problems followed by solutions or alternatives, thus becoming relevant topics for the actors in the political process (Dunn, 1994; Kingdon, 1995).

^3^ https://formiche.net/2022/08/russia-interferenze-italia-copasir/

^4^ https://www.agcom.it/documents/10179/28529091/Studio-Ricerca$+$02-11-2022/d5e11837-cedc-49ed-9b09-c1f28e59fe31?version=1.1
