# Supplementary material for: The SEO effect. Mapping the optimized landscape around controversial policy issues in Italy
Source: Front Sociol. 2023 Feb 23;8:1144669. doi: 10.3389/fsoc.2023.1144669 (PMC9996319; doi:10.3389/fsoc.2023.1144669)
Supplement: Supplementary file 1 [file Data_Sheet_1.PDF]

Topic	Keywords
	aiutiamo i lavoratori
	ammortizzatori sociali
	basta lavoro nero
	basta lavoro povero
	basta precariato
	bonus edilizi
	caro vita
	cassa integrazione per professionisti e autonomi
	centralità lavoro
	colmare diseguaglianze
	combattere burocrazia
	combattere precarietà
	consentire di trovare lavoro a percettori reddito
	contrasto lavoro irregolare
	contrasto precariariato
	decontribuzione lavoro femminile
	decontribuzione sud
	decreto dignità
	detassare premi produttività
	difesa made in italy
	disincentivo part time involontario
	eliminare reddito di cittadinanza dopo rifiuto
	equiparazione congedo paternità e maternità
	equo compenso
	estensione voucher lavoro
	facilitazione accesso al credito
	flessibilità accesso pensione
	incentivare crescita studi professionali
Work	incentivazione welfare aziendale

	incentivi imprenditoria femminile
	integrazione pubblica alla retribuzione
	interventi sull'iva calmierare prezzi
	lavoratori autonomi partecipazione bandi
	lavoro
	lotta al lavoro nero e sommerso
	lotta al precariato
	maggiori tutele lavoro autonomo
	mensilizzazione versamento imposte dirette per autonomi
	metadone di stato
	obbligo retribuzione stage
	oppressione fiscale
	parità salariale
	più diritti e più uguaglianza
	promozione smart working
	promuovere flessibilità
	rafforzamento politiche attive lavoro
	rafforzamento sicurezza luoghi di lavoro
	reddito cittadinanza
	riduzione dell’orario di lavoro a parità di salario
	riduzione disuguaglianze
	riduzione iva prodotti energetici
	riforma pensioni
	rinforzo reddito cittadinanza
	salario minimo
	stop stage e tirocini gratuiti
	supportare imprese
	taglio cuneo fiscale per imprese e lavoratori
	abbassare tasse sul lavoro
	abolizione irap

Tax	abolizione micro tasse
	accredito su conto corrente
	cashback fiscale
	cessione credito fiscale
	codice tributario
	conto corrente per tutti i cittadini
	conto unico fiscale
	detassazione giovani
	estensione detrazione irpef startup
	fisco
	fisco digitale
	fisco equo
	fisco progressivo
	flat tax
	incrociamento banche dati
	interventi iva
	liquidazione mensile imposte partite iva e liberi professionisti
	maxi rateizzazazione cartelle esattoriali
	potenziare agenzie fiscali
	potenziare credito d'imposta
	premialità fiscale ambientale
	riduzione iva energia
	riduzione pressione fiscale
	riduzione tassazione del risparmio
	semplificazione sistema tributario
	semplificazione struttura aliquote
	superbonus
	taglio del cuneo fiscale
	tasse
	tracciabilità pagamento

Migration	abolizione bossi fini
	accoglienza
	accordi cooperaziione
	accordo europeo redistribuzione
	allargamento corridoi umanitari
	combattere immigrazione clandestina
	immigrazione irregolare
	contrasto all'integralismo islamico
	cooperazione
	favorire ingressi regolari e programmati
	immigrazione
	inclusione
	ingresso legale
	integrazione
	italia più umana e sicura
	ius scholae
	ius soli
	migranti economici
	ministero per le migrazioni
	no barriere immigrazione
	no invasione
	no islamizzazione
	no ius soli
	nuova legge immigrazione
	politiche di asilo
	politiche integrazione
	porti aperti
	prima gli italiani
	profughi umanitari
	regolarizzazione migranti irregolari residenti

	richiedenti asilo
	rimpatrio automatico per immigrati irregolari
	rispetto quote nazionali
	stop agli sbarchi
LGBTQIA+	amore fluido
	autorità nazionale indipendente per tutela diritti umani
	diritti persone omosessuali
	diritto famiglie omogenitoriali
	famiglia naturale
	genitore 1 genitore 2
	l'amore non ha genere
	l'amore non ha sesso
	legge contro l’omolesbobitransfobia
	legge contro omotransfobia
	legge unioni civili
	matrimonio egualitario
	no al matrimonio tra persone dello stesso sesso
	no ddl zan
	omolesbobitransofobia
	prevenzione e contrasto linguaggio d'odio
	reato universale
	riconoscimento adozione
	riconoscimento figli
	sconfiggere misoginia
	sì ddl zan
	stop gender
	tutela diritti civili
	tutelare famiglie arcobaleno
	utero in affitto
	utero in affitto reato

Innovation and digitisation	banca dati digitale nazionale
	banda ultralarga
	carta dei diritti digitali
	cittadinanza digitale
	competenze digitali
	connettività diffusa
	copertura reti
	cybersecurity
	digitalizzazione pubblica amministrazione
	digital literacy
	digitalizzazione
	digitalizzazione catasto
	digitalizzazione del patrimonio culturale
	digitalizzazione del servizio giustizia
	digitalizzazione delle infrastrutture idriche
	diritto alla connessione digitale
	educazione digitale
	etichettatura digitale
	fisco digitale
	incentivi startup
	infrastrutture digitali
	innovazione
	lavoro agile
	materie stem
	pubblica amministrazione in cloud
	regolazione applicazioni dell’intelligenza artificial
	sistema di rimborso dei dati dei cittadini
	smart buildings
	space economy
	tecnologie strategiche per il futuro

	territori intelligenti
	transizione 4.0
	transizione digitale imprese
Healthcare	aggiornamento piani di emergenza
	aggiornamento piani pandemici
	assistenza residenziale e domiciliare
	assistenza territoriale
	attività di screening
	aumento stipendi operatori sanitari
	case della comunità
	censimento strutture sanitarie pubbliche e private
	digitalizzazione sistema sanitario
	educazione alimentare
	educazione sessuale
	estensioni prestazioni esenti ticket
	farmacia dei servizi
	fascicolo sanitario elettronico
	fecondazione medicalmente assistita
	filiera innovazione sanitaria
	fondo screening neonatale esteso
	formazione medici e personale sanitario
	incremento organico di medici e operatori sanitari
	infermieri di comunità
	medicina ambientale
	medicina di genere
	medicina predittiva
	medicina territoriale
	nuovo sistema lea
	liste d'attesa
	piano salute mentale

	prevenzione
	protezione civile sanitaria
	psicologo di base
	medicina generale
	revisione piano oncologico
	revisione piano sanitario nazionale
	riconoscimento caregiver
	riforma sulla non autosufficienza
	riordino scuole specializzazione
	sanità
	sostegno alla fecondazione
	sport
	sviluppo sanità di prossimità
	telemedicina
	risparmio energetico
	ambiente
	completamento della carta geologica
	comunità energetiche
	cambiamenti climatici
	economia circolare
	economia del mare
	educazione ambientale
	energia pulita
	energie rinnovabili
	incentivare l'utilizzo del trasporto pubblico
	legge quadro sul clima
	legge sul consumo di suolo
	manutenzione delle foreste
	mix fonti energetiche
	mobilità urbana sostenibile

Environment	obiettivo emissioni zero
	premialità fiscale per le imprese a elevato rating esg
	resilienza delle aree a rischio dissesto idrogeologico
	riciclo dei rifiuti
	riconversione energetica
	ridurre il consumo delle risorse naturali
	ridurre l'impatto del trasporto merci
	riduzione domanda energia
	emissione co2
	rimboschimento e piantumazione
	salvaguardia della biodiversità
	società 2000 watt
	stop a nuove trivellazioni e a nuovi inceneritori
	superbonus energia imprese
	tecnologie green
	transizione ecologica
	trasformare rifiuti in energia rinnovabile
	tutela biodiversità
	tutela e la salvaguardia della qualità delle acque
Abortion	aborto
	cimitero feti
	difesa del diritto all'aborto
	diritti umani donne
	diritto di abortire
	interruzione volontaria gravidanza
	legge 194
	obiettori di coscienza
	omicidio feto
	pro aborto
	pro life

Euthanasia	diritto all'eutanasia
	diritto di scegliere fine vita
	eutanasia
	fine vita
	liberi fino alla fine
	morte volontaria medicalmente assistita
	omicidio consenziente
	sacralità della vita
	tutela della vita
